# The potential of CT colonography for colorectal cancer screening in Japan

**DOI:** 10.1007/s11604-025-01798-2

**Published:** 2025-05-10

**Authors:** Kenichi Utano, Masato Aizawa, Noriyuki Isohata, Yuka Utano, Shungo Endo, Kazutomo Togashi

**Affiliations:** 1 Department of Radiology, Aizu Medical Center, Fukushima Medical University, 21-2 Maeda, Tanisawa, Kawahigashi, Aizuwakamatsu, Fukushima 969-3492 Japan; 2 Department of Coloproctology, Aizu Medical Center, Fukushima Medical University, Fukushima, Japan

**Keywords:** CT colonography, Mass screening, Colorectal neoplasms, Quality control

## Abstract

Colorectal cancer remains a leading cause of mortality worldwide, and early detection is essential for improving outcomes. CT colonography (CTC) has emerged as a promising alternative to optical colonoscopy for colorectal cancer screening. This article explores the potential of CTC in Japan, focusing on quality control, patient acceptability, complications, and its role in screening programs. CTC has demonstrated high sensitivity and specificity for detecting colorectal polyps, with its diagnostic performance comparable to colonoscopy for lesions ≥ 10 mm. Techniques such as fecal tagging and dual-position imaging significantly enhance diagnostic accuracy. However, the variability in diagnostic outcomes underscores the need for rigorous interpretation training and quality control. The American College of Radiology recommends training with at least 50 cases verified by colonoscopy. Despite its advantages, the adoption of CTC in Japan remains limited due to low awareness among medical professionals, a shortage of trained radiologists, and the absence of specific guidelines endorsing its use. Patient acceptability for CTC is high due to its non-invasive nature, shorter examination time, and reduced bowel preparation requirements compared to colonoscopy. Nonetheless, complications such as bowel perforation, albeit rare, necessitate careful risk assessment. While CTC has been recognized in the U.S. and Europe for screening and diagnostic follow-up, its integration into Japan’s colorectal cancer screening guidelines is crucial to expand its utilization. To maximize the benefits of CTC, efforts must focus on standardizing methodologies, establishing quality indicators, and generating robust evidence on mortality reduction and cost-effectiveness.

## Introduction

Colorectal cancer remains a leading cause of mortality worldwide, making early detection essential for improving outcomes. Recently, CT colonography (CTC) has emerged as a promising alternative for colorectal cancer screening. CTC has demonstrated high sensitivity and specificity for detecting colorectal polyps, with its diagnostic performance nearing that of colonoscopy for lesions ≥ 10 mm. Despite its advantages, the adoption of CTC in Japan remains limited due to low awareness among medical professionals, a shortage of trained radiologists, and the absence of specific guidelines endorsing its use. This article explores the critical aspects of CTC, including quality control, patient acceptability, potential complications, and its role in cancer screening. Additionally, it provides an overview of the current status of colorectal cancer screening in Japan and the potential of CTC for improving screening practices.

### Importance of quality control in CT Colonography

Colonoscopy is widely recognized, both in Japan and globally, as the most accurate method for diagnosing colorectal polyps. Conversely, CTC has seen limited use in clinical practice, primarily due to concerns over its significantly lower polyp detection rates compared to colonoscopy. This concern stems from discrepancies in polyp detection rates reported in three clinical trials from the early 2000 s, each including 500 to 1,000 participants [1–3]. Analyses of these studies highlighted essential factors for ensuring the accuracy of CTC, including fecal tagging, adequate reading training, dual-position imaging, and 3D image interpretation using dedicated workstations.

The American College of Radiology Imaging Network, an organization affiliated with the United States (US) government, allocated approximately 6 million US dollars for a large-scale trial comparing colonoscopy and CTC accuracy. This trial included 2531 participants across 15 facilities in the US. Published in 2008, this landmark trial demonstrated that CTC sensitivity for detecting adenomatous polyps ≥ 10 mm and ≥ 6 mm was 90% and 78%, respectively, nearly matching colonoscopy [4]. Later large-scale trials consistently reported sensitivities over 90% for lesions ≥ 10 mm, further validating its efficacy [5–10] (Table 1). These findings have established CTC as an effective screening tool for colorectal cancer, leading to its inclusion in several US screening guidelines [11]. Significant progress in radiation dose reduction has been achieved in recent years, driven by enhancements in CT hardware capabilities and the implementation of advanced iterative reconstruction algorithms, such as model-based or hybrid techniques.

**Table 1:** Clinical trials on CT colonography

Lead Author	Pickhardt [1]	Cotton [2]	Rockey [3]	Johnson [4]	Zalis [5]	Graser [6]	Regge [7]	Heresbach [8]	Nagata [9]	Utano [10]
Country	United States	United States	United States	United States	United States	German	Italia	Fance	Japan	Japan
Publication year	2003	2004	2005	2008	2012	2009	2009	2011	2017	2017
Number of Cases	1253	615	614	2531	694	311	1103	845	1260	321
Use of tagging	Yes	No	No	Yes	Yes	Yes	Yes	Yes	Yes	Yes
Interpretation method	Fly-through	Fly-through	Unknown	Fly-through	Fly-through	Fly-through	Fly-through	Fly-through	Fly-through	Fly-through
Per-patient sensitivity (Lesion ≥ 10 mm)	94%	55%	59%	90%	91%	92%	90%	75–92%	91–93%	91%
Per-patient specificity (Lesion ≥ 10 mm)	96%	96%	96%	86%	85%	98%	84%	95–98%	98–99%	99%

### Key components of quality control in CT colonography

#### Fecal tagging

**Fig. 1:**
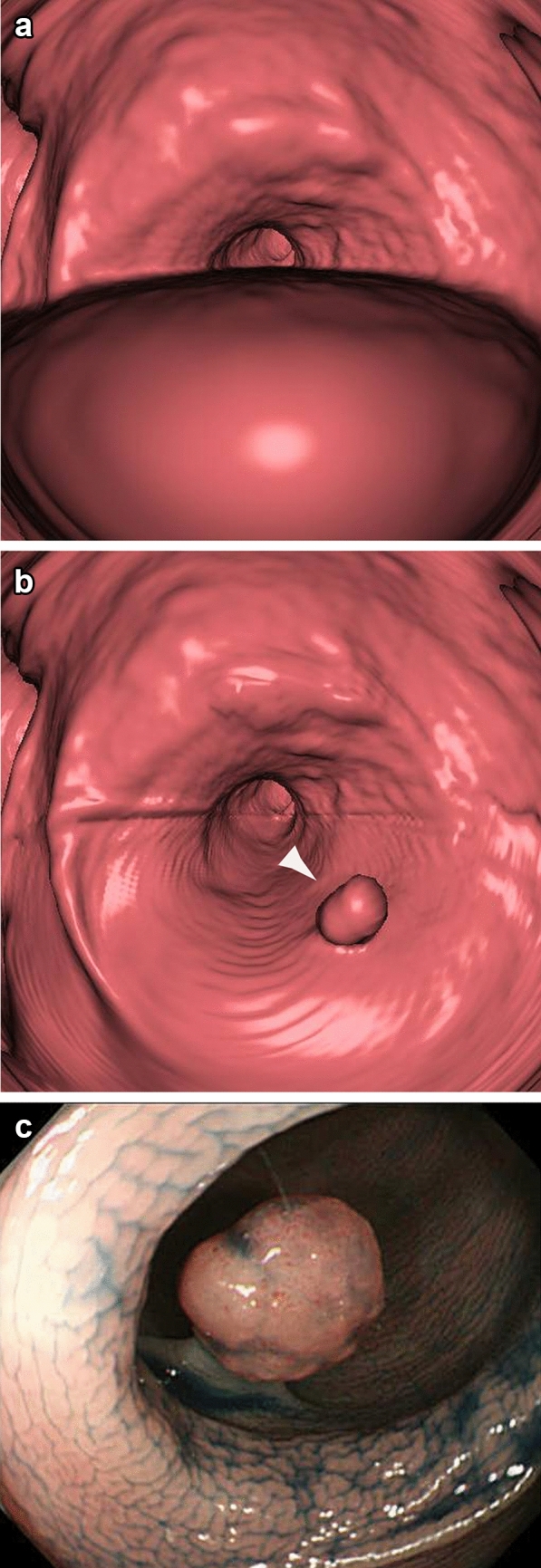
**a** On standard fly-through imaging, submersed lesions are typically obscured and not visualized. **b** Following the application of electronic cleansing, the tagged residual fluid was effectively removed, enabling clear identification of the lesion (►). **c** Endoscopic view of the lesion, which was subsequently resected and pathologically confirmed as an 8-mm adenoma

**Fig. 2:**
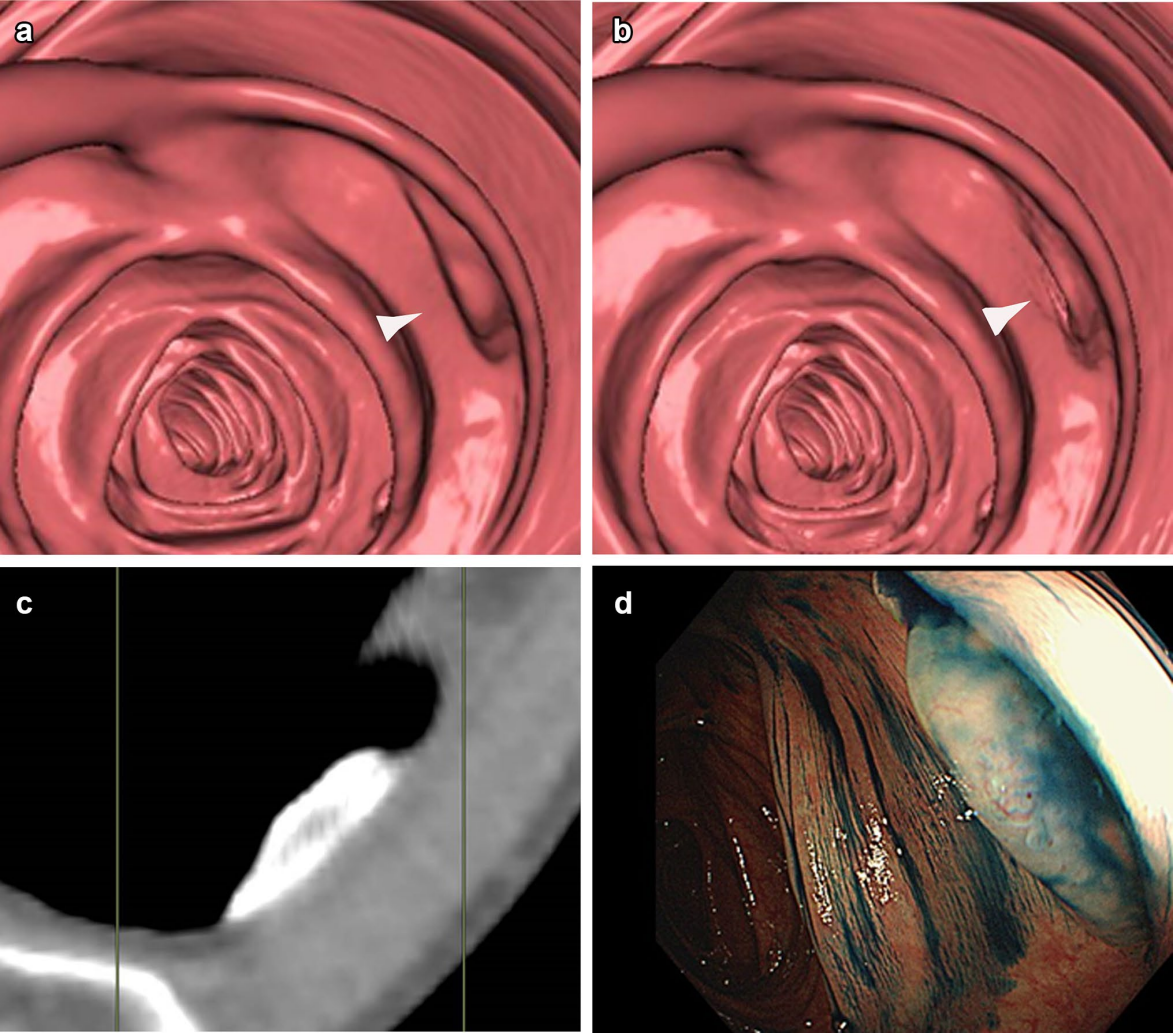
Sessile Serrated Lesion (SSL), which has recently attracted attention as the third pathway of carcinogenesis, often presents as a flat lesion with mucus attached to its surface. **a** A 57-year-old female underwent CT colonography following a positive screening result, revealing a 15 mm type II a lesion in the ascending colon. ► identifies the sessile serrated lesion. **b** When electronic cleansing was applied, the lesion disappeared (►). **c** On the multi-planar reconstruction image, the lesion appeared to resemble tagged mucus. **d** An endoscopic image of the same lesion. The pathological diagnosis was SSL

Fecal tagging in CTC involves using oral contrast agents, such as barium or diatrizoate meglumine, to label residual bowel materials. In contrast to colonoscopy, CTC lacks the ability to suction or remove residual liquid and stool from the colon. Moreover, the commonly used"fly-through"viewing method cannot visualize the interior of liquid-filled areas. Fecal tagging addresses these limitations. Ingesting a small amount of oral contrast agent before CTC increases the CT values of residual materials and liquid in the colon, facilitating their distinction from polyps and significantly enhancing diagnostic accuracy. CTC with tagging eliminates the need for complete bowel cleansing, greatly reducing the amount of preparation agents required [12].'Electronic cleansing'has also been developed to remove tagged residual materials and liquid from workstation images, theoretically enabling near-perfect visualization of the colon (Fig. 1). However, the effectiveness of electronic cleansing varies depending on the workstation, and there is a risk of inadvertently removing polyps with adhered mucus, necessitating cautious application of this technique (Fig. 2).

Without fecal tagging, CTC struggles to differentiate residual matter from lesions, resulting in reduced diagnostic accuracy. Consequently, fecal tagging is indispensable for both primary screening and detailed examinations of individuals with positive screening results. By eliminating the need for complete bowel cleansing, fecal tagging significantly reduces the required amount of bowel preparation agents. In 2012, Zalis et al. reported favorable outcomes for CTC when combining computer-aided detection and electronic cleansing, achieving accurate results without the use of bowel preparation agents or laxatives [5]. Since colonoscopy necessitate the ingestion of large quantities of bowel cleansing agents、this study effectively highlighted a major advantage of CTC over colonoscopy. In Japan, only barium‐based oral tagging agents are currently approved for clinical use and covered under national insurance, which limits the choice of contrast materials compared with some Western countries.

#### Interpretation training

The ability of CTC to visualize colorectal neoplastic lesions is generally considered high; however, significant variability in diagnostic accuracy has been reported in clinical trials [1–3]. Among the factors contributing to this variability, reader proficiency is particularly critical. Therefore, adequate training is essential for accurate interpretation of CTC results.

The American College of Radiology guidelines recommend completing interpretation training with at least 50 cases confirmed by colonoscopy before independently interpreting CTC [13]. Previous studies have estimated that a novice in abdominal CT interpretation needs to review approximately 164 cases to achieve adequate proficiency, with formal training programs often requiring 175 cases [14]. Crucially, training cases must include lesions confirmed or excluded via colonoscopy; otherwise, readers risk failing to detect subtle lesions that are easily missed in CTC. For experienced readers, the time required to interpret each case is relatively short. For example, the JANCT study reported an average interpretation time of 9.97 min per case [9], while the UMIN6665 study recorded an average of 10.6 min per case [10].

As CTC becomes more widely adopted, the shortage of radiologists in Japan—where their numbers are among the lowest among OECD member countries—has necessitated the involvement of radiological technologists in initial interpretations [15]. A meta-analysis of radiological technologists'interpretations reported a sensitivity of 76% and a specificity of 74% on a per-patient basis. Although current evidence is insufficient to support independent interpretation by technologists, the study concluded that proper training could lead to significant improvements [16].

In addition to its primary use, CTC can evaluate extracolonic organs due to its abdominal imaging capability [17, 18]. However, this raises concerns about detecting numerous clinically insignificant findings. The US Preventive Services Task Force has acknowledged both the benefits and drawbacks of identifying extracolonic findings [19]. Similarly, the NCCN guidelines for colorectal cancer screening have emphasized concerns regarding the detection of findings that do not require follow-up [20]. Nevertheless, we occasionally detected significant lesions on CTC, such as asymptomatic malignancies, which could be clinically meaningful for patients.

#### Imaging

CTC is conducted following bowel preparation and rectal insufflation of gas, typically carbon dioxide. The imaging process relies on the significant CT value difference between the expanded intestinal lumen and the bowel wall to visualize the colon. Inadequate bowel distension or preparation can markedly reduce diagnostic accuracy, making bowel distension a critical factor for high-quality imaging. Optimal imaging requires adequate bowel preparation to minimize residual stool and fully distend the colon. If one or more segments of the colon remain collapsed in both positions, evaluation becomes impossible, necessitating further assessment through alternative diagnostic methods [21]. Automated CO2 insufflation devices have been shown to improve patient acceptance and ensure consistent bowel expansion by delivering gas at a constant pressure [22]. While the use of antispasmodics during imaging does not typically affect bowel distension [23], it may be considered for patients with strong peristalsis.

In practice, comparing two imaging positions is necessary to distinguish lesions from residual stool, typically involving prone and supine positions. In certain cases, lateral positioning may also be acceptable [24]. Since residual stool shifts with changes in body position, imaging in at least two positions facilitates differentiation between lesions and stool. Combining tagging techniques with positional changes enhances diagnostic accuracy and minimizes the risk of missed lesions.

Since CTC is intended as a repeated screening examination conducted every few years, careful consideration must be given to medical radiation exposure. The requirement for imaging in two positions makes radiation reduction even more critical compared to standard abdominal CT imaging. Radiation dose can be minimized by lowering the tube current and voltage, as well as by reducing the frequency of imaging. The radiation dose is directly proportional to the tube current and inversely proportional to the square of the tube voltage. Although CTC is commonly performed at a standard tube voltage of 120 kilovolt peak, recent studies suggest that reducing the tube voltage to approximately 100 kilovolt peak effectively lowers radiation exposure [25]. CTC takes advantage of the significant CT value difference (approximately 1000 Hounsfield unit) between the CO2-expanded bowel lumen and the bowel wall, enabling substantial dose reduction. Imaging is recommended at a tube current of 50 milliampere-second or less, or with the use of automatic exposure control [26]. According to European consensus, the average effective dose per CTC examination should be limited to approximately 3–5.7 milli sievert [27], while the American College of Radiology guidelines recommend a total absorbed dose not exceeding 12.5 milli sievert per examination [13]. In earlier CT imaging, excessive dose reduction often led to increased noise and degraded image quality, complicating the interpretation of ultra-low-dose CTC. However, with the development of advanced reconstruction methods, such as iterative reconstruction techniques, it is now possible to significantly reduce noise even in ultra-low-dose imaging. Nonetheless, caution is required, as ultra-low-dose imaging with radiation exposure below 1 mSv may compromise diagnostic accuracy [28].

### Acceptability of CT colonography

CTC does not require endoscopic insertion or the use of barium enemas, and its relatively short examination time is generally regarded as an advantage, as it places less burden on patients. Furthermore, the procedure does not demand the high level of expertise required for colonoscopy and is unaffected by patient-specific factors such as gender, age, body size, or a history of abdominal surgery. However, when polyps 10 mm or larger are detected, a subsequently colonoscopy becomes necessary, and the sensitivity of CTC for detecting flat lesions is lower compared to elevated lesions [29, 30]. In a prospective study conducted in Japan involving approximately 900 individuals with positive screening results, 28% chose CTC, while 72% opted for colonoscopy. The primary reason for choosing colonoscopy was its capability to remove polyps during the procedure, whereas the main reason for selecting CTC was the perception of reduced discomfort. Post-procedure surveys revealed that the most uncomfortable aspect of CTC was gas insufflation, while, surprisingly, the most common response for colonoscopy was “no significant discomfort,” with gas insufflation being the second most reported issue [31]. Recent advancements in endoscopic technology have significantly improved imaging quality, even with thinner endoscopes, thereby reducing the discomfort associated with colonoscopy. Additionally, studies suggest that colonoscopy performed under intravenous sedation offers higher patient acceptability compared to CTC [32]. At present, patients may experience the least discomfort during sedated colonoscopy.

### Complications of CT colonography

A serious complication associated with CTC is bowel perforation. Risk factors include severe colorectal cancer-related strictures, advanced diverticulosis, inflammatory bowel disease, and inguinal hernias involving sigmoid colon incarceration [33]. Documented cases of perforation include rectal perforation caused by a rectal catheter used for contrast enema and sigmoid colon perforation due to diverticula [34]. A 2014 meta-analysis on CTC-related complications reported an incidence of bowel perforation at 0.027% (28 out of 103,399 cases) [35]. Similarly, a 2017 national survey in Japan covering over 140,000 CTC cases reported a perforation rate of 0.014% (21 out of 147,439 cases). Among the 21 perforation cases, four required surgical intervention (0.003%), while the remaining 17 were successfully treated conservatively [36]. CTC has the capability to detect free air with high sensitivity, making it likely that even extremely small perforations can be accurately diagnosed.

**Fig. 3:**
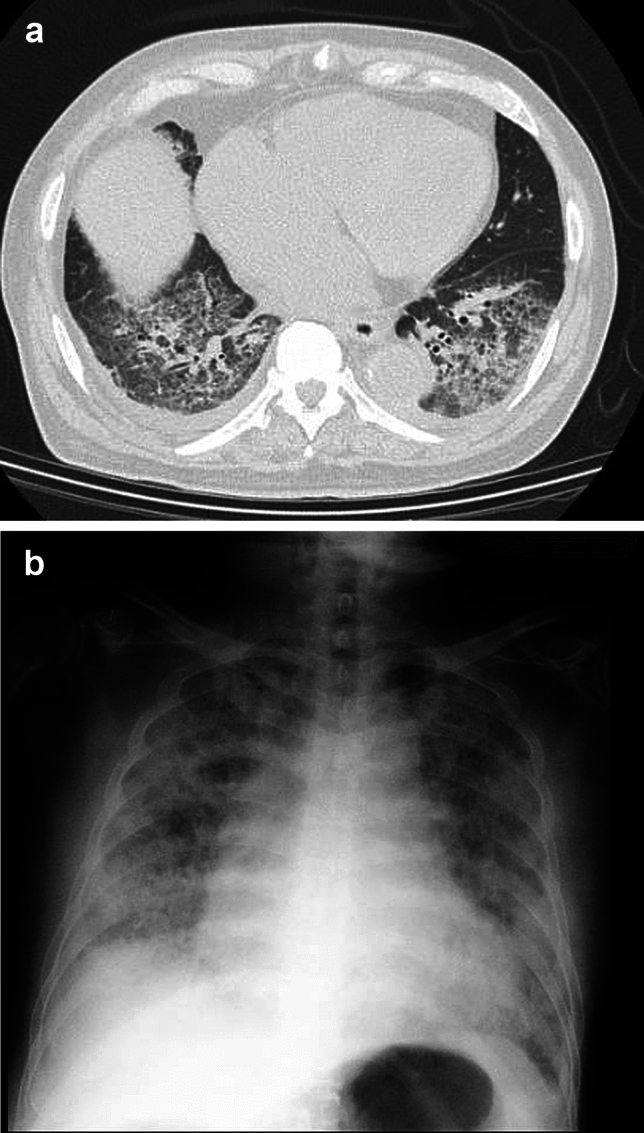
A 90-year-old male was scheduled for CT colonography as part of a detailed examination for abdominal pain. Bowel preparation included Diatrizoate meglumine and a bowel cleansing agent. On the day of the examination, the patient developed respiratory failure and was urgently hospitalized. **a** A chest CT scan taken on the same day revealed pneumonia in both lower lobes of the lungs, leading to a diagnosis of aspiration pneumonia caused by the bowel preparation agents. **b** A plain chest X-ray taken two days later showed significant worsening of the pneumonia. Despite intensive care, including steroid pulse therapy, the patient died on that day

**Fig. 4:**
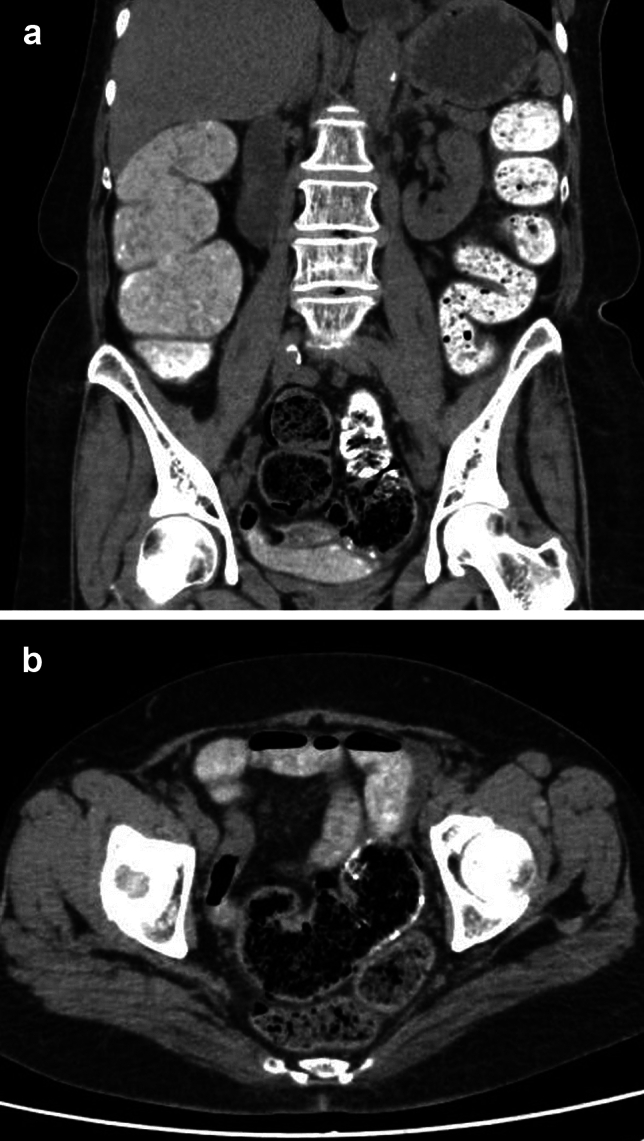
A 66-year-old female was scheduled for CT colonography to investigate the cause of her constipation. She took a bowel preparation agent containing barium starting the day before the examination, but no bowel movements occurred. On the day of the examination, she presented with fever, abdominal pain, and low blood pressure, entering a state of shock and requiring hospitalization. **a, b** Tagged stool was observed from the ascending colon through the sigmoid colon, while untagged stool was found in the rectum. The patient was diagnosed with sepsis secondary to obstructive colitis and died the following day

To date, no mortality directly linked to CTC has been reported, reinforcing the belief that it is a highly safe procedure. However, this absence of reports may be due to publication bias. Unfortunately, at our institution, we have encountered two cases of procedure-related deaths (Figs. 3 and 4). These cases constitute highly specific events observed in super-elderly patients at a rural medical facility in Japan and are not deemed to materially affect the established safety profile of CTC. Nevertheless, these incidents underscore the inherent risks of severe complications associated with CTC, particularly due to bowel preparation and severe constipation. Overconfidence in the safety of CTC can be misleading, and caution must always be exercised.

### Current status of colorectal cancer screening in Japan

In Japan, the number of deaths from colorectal cancer has remained at a persistent plateau in recent years. Projections for 2023 estimated 53,000 deaths from colorectal cancer, making it the second leading cause of cancer-related mortality after lung cancer, which accounted for 75,700 deaths [37]. In contrast, colorectal cancer deaths in the US have been gradually declining, with a projected 52,550 deaths in 2023 [38]. Considering that Japan's population is approximately 40% of the US, the high number of colorectal cancer deaths in Japan, even in the context of an aging population, is alarming. Screening is an established method to reduce colorectal cancer mortality, with fecal immunochemical testing (FIT) demonstrating its effectiveness through multiple randomized controlled trials (RCTs) [39, 40]. According to the 2022 Comprehensive Survey of Living Conditions in Japan, the colorectal cancer screening rate was 45.9% (49.1% for men and 42.8% for women) [41]. Although this represents a gradual upward trend, it still falls short of the 50% target established by the Basic Plan to Promote Cancer Control Programs in 2007. Additionally, the low rate of follow-up diagnostic testing for primary screening-positive individuals remains a significant issue. The 2020 Regional Health and Health Promotion Project Report showed that the follow-up rate for colorectal cancer was 69.9%, the lowest among cancers such as gastric cancer (84.4%), lung cancer (82.5%), cervical cancer (77.6%), and breast cancer (89.9%) [42]. A possible explanation for this is that colonoscopy is the only available option for a detailed examination, and a certain proportion of individuals may be reluctant to undergo the procedure. Under these circumstances, there is increasing support for the active use of highly accurate CTC in colorectal cancer screening. In 2016, the Japanese Society of Gastroenterological Cancer Screening issued a committee report recommending CTC or a combination of sigmoidoscopy and barium enema as reasonable alternatives in situations where total colonoscopy is challenging as a follow-up diagnostic test [43].

Colorectal cancer screening in Japan targets individuals aged 40 and older, with annual FIT being the standard recommendation. According to the 2021 Regional Health and Health Promotion Project Report, 3,536,875 individuals underwent colorectal cancer screening, of whom 192,536 tested positives. The follow-up rate for diagnostic testing was 69.9%, and 5,479 individuals were diagnosed with colorectal cancer, representing 0.15% of all screened participants and 2.85% of those who underwent follow-up diagnostic tests [42]. In other words, one in 660 screening participants and one in 35 individuals undergoing diagnostic follow-up are diagnosed with colorectal cancer.

### Effectiveness of various screening modalities for colorectal cancer

The National Cancer Institute in the US reports that colorectal cancer screening tools, such as FIT and sigmoidoscopy, have demonstrated reductions in mortality rates of 15–33% and 22–31%, respectively, based on RCTs with strong internal validity. In contrast, colonoscopy lacks RCT-based evidence of its effectiveness in reducing mortality, relying primarily on observational and case–control studies, which weakens its internal validity [44]. To address this issue, five RCTs are currently being conducted, both domestically and internationally, to evaluate the effectiveness of colonoscopy for colorectal cancer screening [45–49]. In 2022, an RCT on colonoscopy-based screening for colorectal cancer, involving 85,000 participants in a 10-year follow-up study, reported no significant reduction in colorectal cancer mortality in the colonoscopy group compared to the control group. This suboptimal result is believed to be attributed to the low participation rate of 42% for colonoscopy [45]. The results of the remaining four RCTs are also attracting attention [46–49].

Similarly, CTC lacks sufficient evidence regarding its mortality reduction rate or cost-effectiveness. Currently, using CTC as a screening tool for asymptomatic individuals—requiring approximately 600 individuals to be screened to detect one case of colorectal cancer—remains inadequately supported by evidence. An RCT comparing FIT and CTC for colorectal cancer screening revealed in an intention-to-treat analysis that the detection rate of advanced neoplasia with CTC was lower than that of FIT. This was attributed to the low participation rate of only 26.7% for CTC [50]. These findings emphasize the importance of accessibility and acceptability in screening programs. High diagnostic accuracy alone may not be sufficient to make a modality suitable for widespread screening use.

On the other hand, CTC has demonstrated high effectiveness as a follow-up diagnostic tool for positive screening results, owing to its high sensitivity and specificity. Notably, its triage capability is remarkable: setting the cutoff value for lesions at 10 mm can reduce colonoscopy requirements by 86%, while a cutoff of 6 mm achieves a 71% reduction [10]. This approach not only shortens colonoscopy waiting times but also allows resources to be reallocated for therapeutic purposes. A 2012 RCT conducted in the Netherlands involving 8,844 participants found no significant difference in the detection rates of colorectal neoplastic lesions ≥ 10 mm between CTC and colonoscopy in an intention-to-treat analysis. However, a trend toward a higher detection rate with CTC was reported [51].

### Current status of CT colonography in Japan

**Fig. 5:**
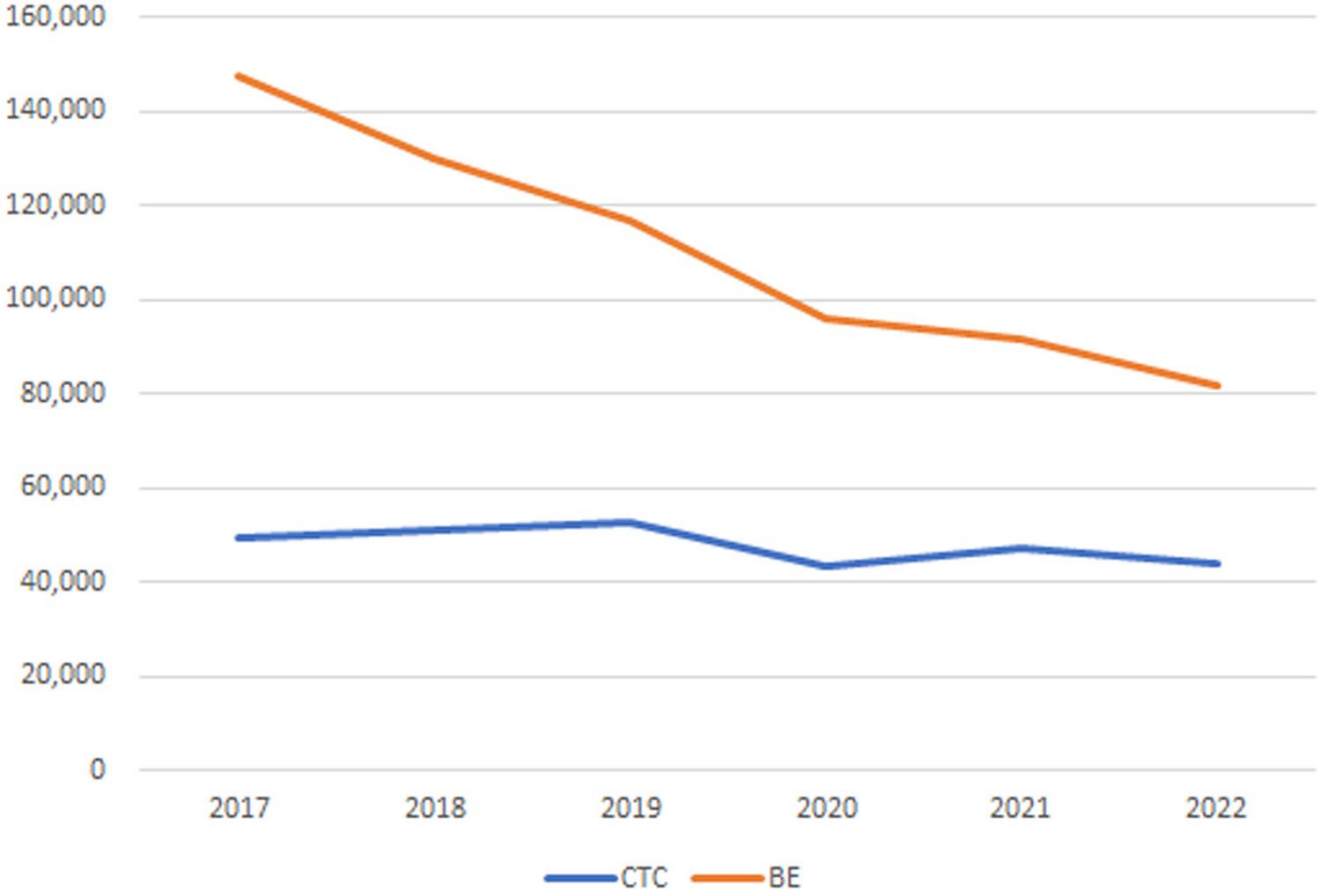
The total number of colorectal examinations conducted nationwide (including both outpatient and inpatient) based on insurance claims. *CTC* CT Colonography, *BE* Barium Enema

An analysis of the National Database of Japan revealed a decline in the number of CTC insurance claims, which had been steadily increasing until 2019 but dropped below 50,000 cases in 2020 due to the COVID-19 pandemic. Meanwhile, although the number of barium enema examinations has been declining, it remained approximately twice that of CTC in 2022 (Fig. 5). The stagnation in CTC usage can be attributed to several factors, including low awareness among medical professionals, a shortage of trained radiologists, and insufficient adoption by healthcare institutions. The utilization of Artificial Intelligence has the potential to mitigate the shortage of radiologists [52]. While CTC is recognized in the U.S. as a screening modality [11, 19, 20] and in Europe as a diagnostic follow-up option when colonoscopy is difficult [27], Japan's “Guidelines for Priority Health Education and Cancer Screening Implementation” and the recently revised “Guidelines for Evidence-Based Colorectal Cancer Screening” lack specific references to CTC. This omission likely contributes to the limited adoption of CTC in clinical practice.

In conclusion, CTC has demonstrated high sensitivity and specificity for detecting colorectal polyps and holds significant promise as an effective tool in Japan, where colorectal cancer mortality remains persistently high. Following the 2021 amendment to the Radiologic Technologist Law, qualified radiologic technologists in Japan may now perform CTC examinations independently, representing a key operational advantage over colonoscopy by reducing the need for an on‐site physician and potentially expanding access. However, its current adoption is insufficient. To maximize the utility of CTC, standardizing testing methodologies, ensuring stringent quality control, and promoting its widespread use are essential. Achieving these goals requires explicitly defining the role of CTC in colorectal cancer screening within the “Guidelines for Priority Health Education and Cancer Screening Implementation” and the “Guidelines for Evidence-Based Colorectal Cancer Screening.” Additionally, establishing quality indicators equivalent to those used in colonoscopy, such as cecal intubation rates and adenoma detection rates [53], is crucial. Furthermore, accumulating robust evidence on the mortality reduction effects and cost-effectiveness of CTC is of also importance. Addressing these challenges will likely elevate CTC as a vital option in colorectal cancer screening, contributing significantly to public health improvement. To achieve this, it is increasingly necessary to generate high-quality evidence from Japan in the future.
